# Cancer fusion transcripts with human non-coding RNAs

**DOI:** 10.3389/fonc.2024.1415801

**Published:** 2024-06-11

**Authors:** Tharaa Mohammad, Marianna A. Zolotovskaia, Maria V. Suntsova, Anton A. Buzdin

**Affiliations:** ^1^ Laboratory for Translational and Genomic Bioinformatics, Moscow Center for Advanced Studies, Moscow, Russia; ^2^ Department of Molecular Genetic Technologies, Laboratory of Bioinformatics, Endocrinology Research Center, Moscow, Russia; ^3^ I.M. Sechenov First Moscow State Medical University, Moscow, Russia; ^4^ PathoBiology Group, European Organization for Research and Treatment of Cancer (EORTC), Brussels, Belgium; ^5^ Shemyakin-Ovchinnikov Institute of Bioorganic Chemistry, Moscow, Russia

**Keywords:** chimeric RNAs, cancer, fusion oncogenes, carcinogenesis, chromosomal rearrangements, long non-coding RNA, lncRNA

## Abstract

Cancer chimeric, or fusion, transcripts are thought to most frequently appear due to chromosomal aberrations that combine moieties of unrelated normal genes. When being expressed, this results in chimeric RNAs having upstream and downstream parts relatively to the breakpoint position for the 5’- and 3’-fusion components, respectively. As many other types of cancer mutations, fusion genes can be of either driver or passenger type. The driver fusions may have pivotal roles in malignisation by regulating survival, growth, and proliferation of tumor cells, whereas the passenger fusions most likely have no specific function in cancer. The majority of research on fusion gene formation events is concentrated on identifying fusion proteins through chimeric transcripts. However, contemporary studies evidence that fusion events involving non-coding RNA (ncRNA) genes may also have strong oncogenic potential. In this review we highlight most frequent classes of ncRNAs fusions and summarize current understanding of their functional roles. In many cases, cancer ncRNA fusion can result in altered concentration of the non-coding RNA itself, or it can promote protein expression from the protein-coding fusion moiety. Differential splicing, in turn, can enrich the repertoire of cancer chimeric transcripts, *e.g.* as observed for the fusions of circular RNAs and long non-coding RNAs. These and other ncRNA fusions are being increasingly recognized as cancer biomarkers and even potential therapeutic targets. Finally, we discuss the use of ncRNA fusion genes in the context of cancer detection and therapy.

## 1 Introduction

**Figure 1:**
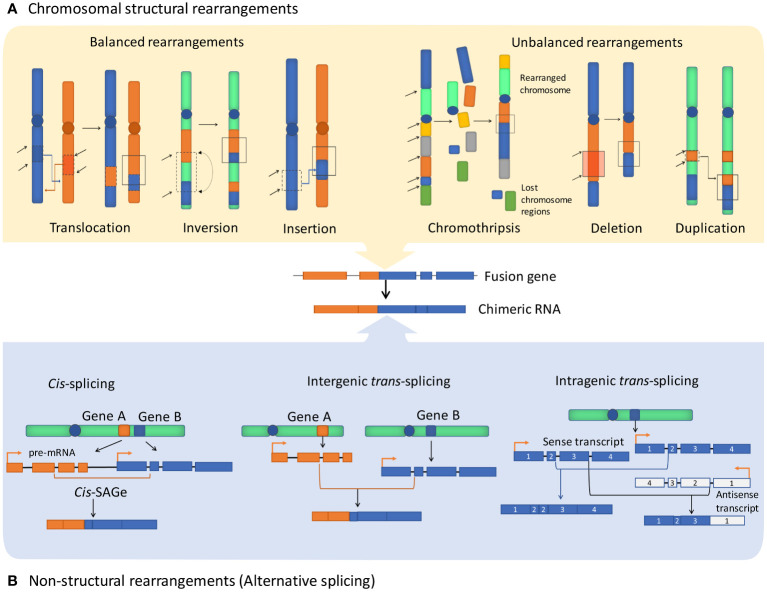
Major fusion genes and chimeric RNA formation mechanisms. Exons and introns are shown as blocks and lines, accordingly. **(A)** Structural rearrangements of chromosomes resulting in the formation of fusion genes include balanced rearrangements that comprise translocations, inversions, and insertions with no loss or gain of genetic material. Unbalanced rearrangements comprise alterations causing extra or missing genetic fragments, including deletions, duplications, and chromothripsis. **(B)** Non-structural rearrangements form non-canonical chimeric RNAs through alternative splicing, without structural rearrangements on the DNA level, including cis-splicing of adjacent genes (cis-SAGe) and trans-splicing, which can potentially involve two different pre-mRNA molecules and can be either intergenic or intragenic.

Cancer chimeric genes, also known as cancer fusion genes, are thought to appear due to chromosomal aberrations that combine moieties of unrelated normal genes. Most commonly, the upstream moiety referred to as 5’ fusion partner retains its promoter and one or group of initial exons, while another moiety provides 3’ terminal part of such chimeric gene (1). The borderline between the two fusion moieties typically located in gene intron sequence is frequently referred to as the fusion breakpoint (2). There are two currently accepted major mechanisms leading to formation of fusion genes. First, structural rearrangements of DNA, either intra- or interchromosomal. This includes translocations, inversions, deletions, insertions, tandem duplications, and chromothripsis (deep rearrangement of multiple chromosomes with loss of many chromosomal segments) (3, 4) (**Figure 1**). Such rearrangements can be either balanced when there is no associated gain or loss of genetic segments or unbalanced which leads to amplifications or deletions in the rearranged chromosomes. Among them, chromosomal translocation is the most commonly mentioned mechanism of gene fusion formation that is strongly associated with many human cancer types (5).

In turn, the second mechanism that is mentioned in the literature in this context comprises generating fusion RNAs without major structural DNA alterations due to splicing or (rather hypothetical) trans-splicing events (**Figure 1**). Chimeric RNAs of this type can be referred to as transcription-induced gene fusions (TIGFs) or transcription-induced chimeras, or “non-canonical chimeric RNAs” (6). These rearrangements may result from splicing and transcriptional errors, such as transcriptional read-through of adjacent genes (7). Overall, chimeric RNAs originated from transcription read-through are referred to as products of cis-splicing of adjacent genes (cis-SAGe) (8). One typical cis-SAGe product is SLC45A3–ELK4 that was found many times in prostate cancer tissues (9, 10). Other examples of cis-SAGe are BCL2L2-PABPN1 and CHFR-GOLGA3 transcripts that are frequently found in human cancers (11, 12), but are also involved in normal and pathogenic adipogenesis in humans and pigs (13, 14) and linked with the Alzheimer disease-precursor phenotypes (15).

Furthermore, trans-splicing of two pre-mRNA molecules potentially can produce trans-TIGFs (7, 16), although no clearly documented cases for such cancer specific aberrations have been currently documented to our knowledge. On one hand, recent developments in deep sequencing enabled high-throughput transcriptome analyses and showed that intergenic splicing could theoretically account for a significant part of chimeric RNAs detected in a pool of RNA sequencing reads (7, 17–19). However, these can represent artifact recombinations by the reverse transcriptase during preparation of the sequencing library. Alternatively, they can relate to genome rearrangement events that could not be detected by the standard approach, e.g. due to their occurrence in the minor fraction of tumor cells.

Finally, events of the recently described fundamental phenomenon of eukaryotic RNA recombination should be also mentioned (20, 21). In this case, different cellular RNAs can be combined when captured and reverse-transcribed by the enzymatic machinery of transposable elements (22, 23). In addition, active families of human transposable elements can fuse to the parts of human genes, thus causing DNA transduction or exon shuffling events (24–26). Note that transposable elements control is strongly disabled in many cancers (27), which can enhance the formation of such chimeras.

The Philadelphia chromosomes discovered in 1960 is one of the best studied types of gene fusion events (28), where a translocation occurs between the chromosomes 9 and 22, thus generating the BCR-ABL fusion gene. Its protein product has constitutively active tyrosine kinase enzyme that promotes carcinogenesis. This fusion is detected in about 95% of chronic myelogenous leukemia (CML) cases, thus making it a hallmark of this disease (29). As a result, CML outcomes have improved dramatically when imatinib and other tyrosine kinase-specific targeted drugs were clinically introduced (18, 30). With the advancement of sequencing technologies, the detection of fusion genes and transcripts was greatly facilitated, and numerous fusions have been identified in various tumor types (31–33). Moreover, some fusion types appeared to be strongly linked with specific cancer types and are considered responsible for roughly 20% of global human cancer morbidity (5, 34). However, most probably the majority of the fusions represent passenger events with no specific function in cancer (35, 36).

In addition, some fusions were also found to be expressed in the normal tissues with no reported oncogenic activity (37). For example, chimeric RNAs of well-known fusions such as JAZF1-JJAZ1 (SUZ12) and PAX3-FOXO1 are sometimes expressed in the normal cells, despite being frequently detected in endometrial stromal sarcomas and alveolar rhabdomyosarcomas, respectively (38–40).

Generally, fusion genes can be classified by their protein coding potentials. They can be capable of coding proteins, where the coding sequence of both fusion partners is in-frame, which gives rise to abnormal proteins with, for example, kinase or transcriptional factor activities (41–45). In addition, a frameshift fusion can occur when both fusion partners normally encode proteins, but the downstream partner open reading frame (ORF) is not in phase with that of the upstream gene (36). And finally, a non-coding fusion is a fusion where the junction site is either outside of the parental gene ORF (out-of-frame fusion), or one or both parental genes are non-coding (37). Here, out-of-frame fusions sometimes can produce novel peptides that share no sequence identity with the original gene products (46). Alternatively, such chimeric transcripts can function as long noncoding RNAs with regulatory functions (47). Interestingly, a recent study found that the vast majority of newly discovered fusion genes in non-translocation-related sarcomas are out-of-frame (8, 48).

Currently, many protein-coding oncogenic fusion genes became clinically actionable, i.e. can serve as the specific targets in tumor molecular therapy (1). As a result, fusions involving non-coding genes attract less attention (49). However, since ncRNAs may play critical roles in transcriptional and post-transcriptional regulation of many genes, they can thereby strongly influence carcinogenesis (50). Many recent studies tried to address fundamental questions regarding the apparent functionality of the non-coding fusion transcripts and the underlying molecular mechanisms (47, 51).

## 2 Classes of ncRNAs and their relation to cancer

Nearly 98% of the human genome is non-protein coding DNA that was previously considered to be “genomic junk” (52, 53). However, most of such non-coding DNA is transcribed (54, 55), and the resulting non-coding transcripts may have various regulatory functions and play a major role in both normal cellular function and pathology, including cancer (50, 56–58).

**Figure 2:**
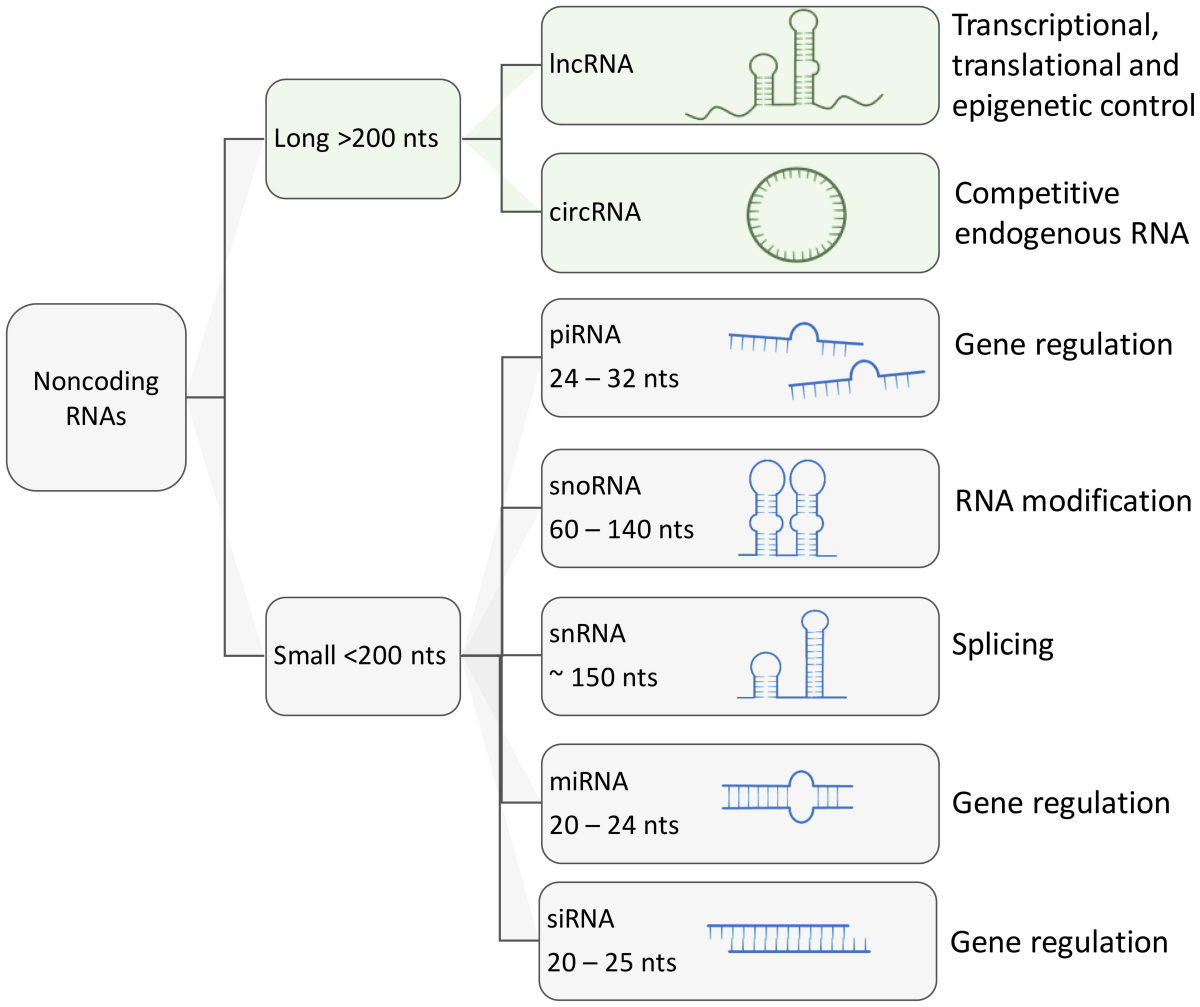
Schematic representation of major types of regulatory ncRNAs and their functions. Aside from housekeeping ncRNA such as transfer RNAs and ribosomal RNAs, other regulatory ncRNAs can be classified by their size. long ncRNAs are larger than 200 nucleotides and can reach up to thousands of nucleotides of length. Small ncRNAs usually are under 200 nucleotides in length, these include small nucleolar RNAs, small nuclear RNAs, PIWI-interacting RNAs, micro RNAs and short interfering RNAs (siRNA).

Based on their length, regulatory ncRNAs can be classified in two major groups: (i) small ncRNAs no longer than 200 nucleotides, including microRNAs (miRNAs), Piwi-associated RNAs (piRNAs), small nuclear RNAs (snRNAs), small nucleolar RNAs (snoRNAs), short interfering RNAs (siRNAs); (ii) longer transcripts that are known as long ncRNAs (lncRNAs) and circular RNAs (circRNAs) (54, 55, 59–64) (**Figure 2**). Overall, major classes of ncRNAs are implicated in the regulation of gene expression in the following ways:

1. lncRNAs: LncRNAs relate to the biggest part of the noncoding transcriptome (65) and are formally classified based on their neighboring protein-coding genes (61). The classes include sense, antisense, intronic, intergenic and bidirectional lncRNAs (66). They are known to play critical roles in the regulation of gene expression at the levels of transcription, RNA stability, and translation, in various biological processes (55, 67). Many lncRNAs can regulate histone modification and structural remodeling of chromatin (68–70), thus contributing to regulation of gene expression and, consequently, cell cycle control and other major cancer related processes (53, 66, 71, 72). In cancers, lncRNAs may exhibit both tumor-suppressive and oncogenic functions (73). Furthermore, expression of biomarker lncRNAs can be utilized to confirm diagnosis and to predict patient outcomes, and several lncRNAs are regarded as promising molecular targets in cancer therapy (70, 74).
2. miRNAs: MicroRNAs (miRNAs) are normally 20 - 24 nucleotides-long regulatory RNA molecules that function by complementary binding to and regulating expression of target mRNAs at the transcriptional and (best studied) post-transcriptional levels (75, 76). A single miRNA can regulate mRNAs of several genes, often hundreds at time (77); in turn, one mRNA can be regulated by several miRNAs spanning complementary sequence to its 3’-untranslated region (78–80). Consequently, the aberrant miRNA expression may influence cancer-related genes and signaling pathways (81). MiRNAs can influence numerous biological processes, including those strongly connected with cancer: proliferation and cell cycle control, differentiation, apoptosis, and migration (81). MiRNAs can be tumor suppressor or oncogenic factors, thus contributing to cancer progression when differentially expressed (82, 83).
3. circRNAs: CircRNAs are ncRNAs that form a circular structure stabilized by covalent bonds. Compared to other cellular RNAs transcribed by RNA polymerase II, circRNAs lack a 5’ end cap and a 3’ end poly-(A) tail and are more stable. Many of them are highly conservative. Some circRNAs act as competitive endogenous RNA (ceRNA), or “molecular sponge” and can suppress function of miRNAs by binding them and, therefore, preventing them from targeting specific protein coding genes (84, 85). CircRNAs have specific profiles in many human pathologies, including cardiovascular diseases (85) and cancers (86–88).
4. snoRNAs: Small nucleolar RNAs (snoRNAs) are typically 60- 300 nucleotides in length and are primarily encoded by intronic regions of protein coding genes or of long non-coding RNAs (89, 90). There are three major groups of snoRNAs: H/ACA box, C/D box, and small Cajal RNAs (scaRNAs) (91). SnoRNAs are abundant in eukaryotic cell nucleoli and play a major role in guiding chemical modifications of ribosomal RNAs (rRNAs) as well as transfer RNAs (tRNAs), mRNAs, and other RNA molecules (90, 92).
5. piRNAs: piRNAs are typically 24–32 nucleotides long noncoding RNAs that bind with P-element-induced wimpy testis (PIWI) proteins (93). Originally, piRNAs were dis-covered in germline cells with their most studied function being control of transposable elements in animal germ cells through complementary binding and targeting degradation of their transcripts (94), thus piRNAs are considered as key regulators for germline maintenance (95).

## 3 Types of fusion gene partners

Fusion events can join both coding and noncoding regions, creating diverse and complex genetic rearrangements. These fusion events can involve various combinations such as coding-coding, coding-noncoding, and noncoding-noncoding gene fusions however, compared to fusion events between protein coding genes, the chimeras joining two ncRNAs or joining ncRNA with protein coding gene represent a relatively underexplored area, and the roles they can play in cancer are still poorly investigated. To a certain extent this may be the consequence of routine filtering out from analytic pipelines of fusion candidates that lack protein coding genes (16). However, efforts have been made to detect and estimate the percentage of fusion transcripts with ncRNA partners including lncRNAs, miRNAs and snoRNAs.

lncRNAs represent one of the most extensively studied classes of ncRNAs (96). Numerous fusion events involving lncRNAs have been reported, with their outcomes largely dependent on the specific positions of the lncRNA partners. While lncRNA-mRNA and lncRNA-lncRNA fusions are likely to remain as transcripts, mRNA-lncRNA fusions can result in the production of proteins with oncogenic functions (97). A pan-cancer bioinformatic study by Guo et al. revealed that lncRNA-lncRNA fusions accounted for over 16 percent of the predicted fusions (98). Despite this prevalence, ncRNA fusions have been less thoroughly investigated for their functional roles in cancer compared to protein-coding gene fusions (50).

Research on breast cancer samples has uncovered a significant involvement of miRNAs and snoRNAs, with 802 pre-miRNA loci encoded in host genes identified as parts of fusion transcripts (99). Furthermore, 780 snoRNA host genes were found to be partners in fusions in at least one breast cancer sample, indicating a selective enrichment of snoRNAs in fusions found in breast cancer samples (100).

## 4 Functions of fusion genes involving ncRNAs in cancer

Additional to their primary regulatory function, cancer-specific mutations and expression level alterations of ncRNAs can strongly influence cell physiology (50). Fusion events have a profound effect on the expression of partner genes, and ncRNAs that are directly involved in fusions can be differentially regulated (47). A correlation between expression of certain lncRNAs, miRNAs, circRNAs, and snoRNAs was detected with specific chromosomal rearrangements in cancers (47, 101, 102).

The effects of fusion events on ncRNAs can be widely versatile. Fusion proteins are known to affect ncRNA in many ways including connecting, amplifying or switching their functions. For example, fusion proteins acting as transcriptional regulators can directly modify expression of ncRNAs (103, 104). In addition, some fusion genes were discovered to act prominently as ncRNA, for example, SLC45A3–ELK4 fusion is an aberrantly spliced chimeric transcript found in prostate cancer. Despite the coding capability of the ELK4 transcription factor moiety (10), SLC45A3–ELK4 fusion drives cancer cell proliferation primarily by acting as long non-coding chimeric RNA (lnccRNA) (105, 106).

**Figure 3:**
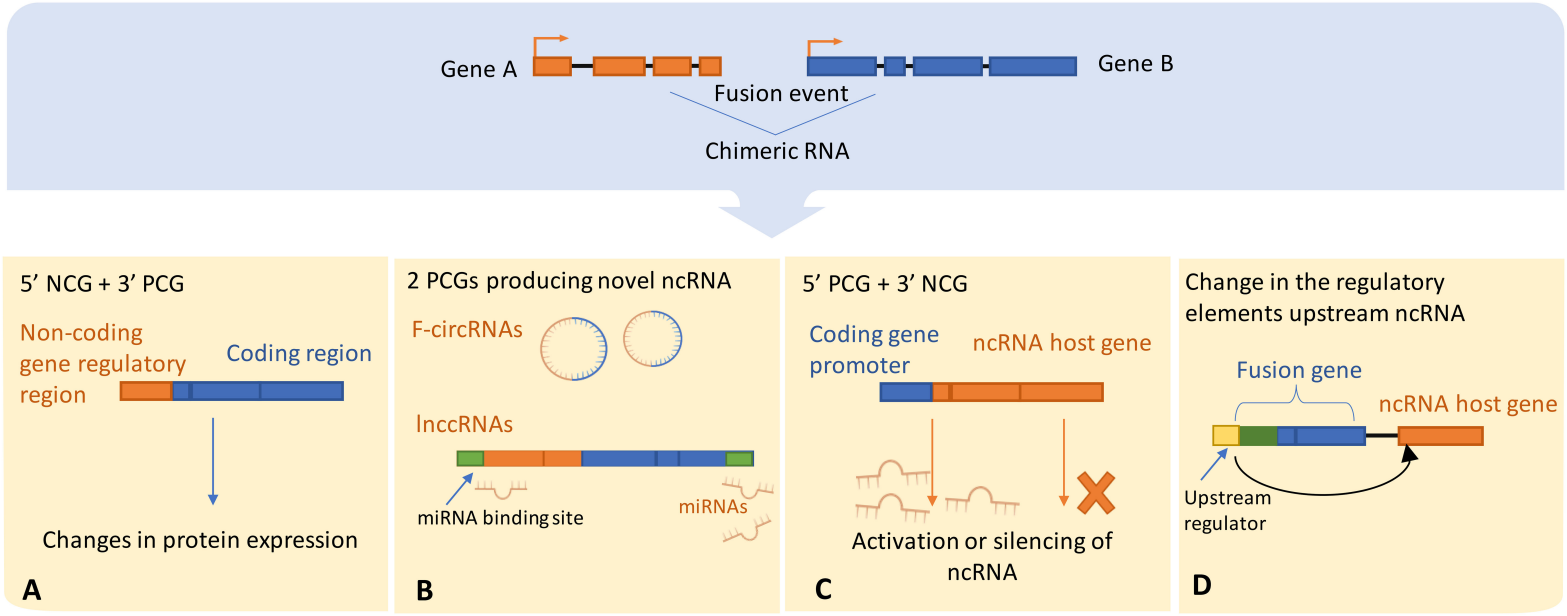
Illustrates possible scenarios by which chromosomal rearrangements involve ncRNAs or directly alter ncRNA expression, **(A)** The regulatory domains of the protein-coding gene may get substituted with the regulatory elements of the ncRNA host gene, thereby leading to dysregulated protein expression. Alternatively, **(B)** The protein-coding gene regulatory regions could either enhance the activation or trigger the silencing of the ncRNA upon exchange with the host gene regulatory elements. ncRNA-convergent fusions may arise when a single ncRNA host gene fuses with diverse genes, establishing a fusion cluster with the purpose of modulating ncRNA expression. **(C)** A fusion event between two protein-coding genes can generate novel chimeric ncRNAs, such as fusion circRNAs and lnccRNAs, that can serve as competitive endogenous RNAs possessing regulatory miRNA binding sites, thereby competing with native transcripts for miRNA binding. **(D)** Ultimately, an alteration in the regulatory domains consequent to a fusion event can lead to downstream dysregulation of ncRNA expression. NCG, Non-coding gene; PCG, Protein coding gene; lnccRNAs, long non-coding chimeric RNAs.

Chromosomal translocations can drive the activation or silencing of ncRNA genes, and many types of ncRNAs are highly susceptible to chromosomal abnormalities. For example, miRNAs were shown to be frequently located in fragile chromosomal regions (107). To our knowledge there are four main scenarios in which chromosomal rearrangements and fusion events can directly influence ncRNAs (**Figure 3**):

- Chromosomal rearrangements joining 5’ non-coding and 3’ protein coding gene moieties can lead to changes in the expression level of truncated protein, or even full protein when the junction site is upstream to the start of the ORF. For example, in MALAT1-GLI1 fusion, part of the lncRNA MALAT gene is translocated into protein coding gene GLI1. This particular fusion was repeatedly found in several cancers including plexiform fibromyxoma, gastroblastoma, and epithelioid neoplasms (108–110),
- Alternatively, a 5’ coding gene and 3’ ncRNA host gene fusion can disrupt the transcriptional control of ncRNAs encoded in introns downstream to the breakpoint. This is a relatively recently discovered type referred to as ncRNA-convergent fusions (99, 111). In their study, Persson et al. discovered that miRNAs were frequent among fusion genes detected in breast cancer. Further, the presence of these miRNAs in fusion genes was non-random, with different 5′ fusion partners independently regulating the expression of the same miRNA, therefore introducing a new class of fusions called “miRNA-convergent fusions” (31, 99, 111). Interestingly, Persson et al. found that there’s no enrichment for properties of protein-coding gene partners in the discovered miRNA-encoding fusions, implying that the function of the miRNA itself, not the potential protein product, provides a selective advantage (99).
- Fusions of two protein coding genes can result in novel chimeric ncRNAs, such as circRNAs and lncRNAs, by alternative or back-splicing. The resulting hybrid molecules can function as regulatory RNAs, examples were reported in both leukemia and solid cancers (102, 112). Examples include the chimeric circRNA derived from MLL-AF9 fusion identified in leukemia, resulting from chromosomal translocation between MLL (Mixed Lineage Leukemia) and AF9 genes. The resulted chimeric circRNA has been shown to play an oncogenic role by promoting the development and progression of leukemia (102). Additionally, coding fusion transcripts can regulate other ncRNAs by functioning in a protein-independent manner. For instance, the most prevalent chromosomal rearrangement in acute myeloid leukemia (AML) resulting in RUNX1-RUNX1T1 fusion transcript was proposed to function as a competing endogenous RNA (ceRNA) that can cross-regulate messenger RNAs by competing for shared miRNAs, that play a crucial role in gene regulation. This competition can impact the level of miRNA available to bind with their mRNA targets, influencing mRNA degradation and subsequently altering gene expression, hence contributing to cancer development (113).
- Chromosomal rearrangements can lead to the exchange of upstream regulatory elements of ncRNAs, hence directly altering its production by either activating or silencing its expression. In AML and myelodysplastic syndrome (MDS), cases with chromosomal translocation t (2, 11)(p21;q23) were connected to overexpression of miR-125b-1 (114). In contrast, the silencing of miR-29 has been reported to coincide with fusion of BCL6 to a non-coding region (115, 116).

### 4.1 Fusions with lncRNA and their functions

Fusion between a lncRNA and a coding gene may have two major implications, both of which affect the coding gene. Other than having a putatively oncogenic function itself, lncRNA involved in a fusion may cause aberrant transcriptional or post-transcriptional regulation of its fusion partner moiety. Either by promoter swapping resulting in the deregulated expression of the 3′ protein-coding gene by the lncRNA promoter, or by truncation of the original ORF causing modification or complete or partial loss of protein function (49). However, ORF truncation of the coding gene can cause loss of protein function, for example in AML frequent gene fusion events with lncRNA genes, such as UBL7-AS1 were reported to cause truncation and loss of function of the RUNX1 encoded transcript, which is a key transcriptional factor in hematopoiesis (117). Overall, most frequently found lncRNA fusions in cancers involved MALAT1 and PVT1 parts (49, 118). Other documented lncRNA fusions also in-volved HOTTIP (119), PCAT1 (120), NEAT1 (121) and Breast Cancer Anti-Estrogen Resistance 4 (BCAR4) (122, 123) ncRNA moieties. Certain lncRNAs, such as MALAT1 and NEAT1, are broadly expressed in the human body (124). However, the majority of lncRNAs are tissue-specific (125), accordingly, such lncRNA-containing fusions can be related to specific cancer types.

#### 4.1.1 Transcriptional regulation:

- **MALAT1 fusions:** Metastasis Associated Lung Adenocarcinoma Transcript 1 (MALAT1), also known as Nuclear-Enriched Abundant Transcript 2 (NEAT2) is a lncRNA localized at nuclear domains known as nuclear speckles (126, 127). Two major known functions of MALAT1 are control of alternative splicing and of transcriptional regulation (118). For example, MALAT1 can regulate expression of the androgen receptor and thereby controls proliferation, invasion, and migration of prostate cancer cells. In addition, MALAT1 is believed to downregulate specific types of splicing in patients with metastatic Castrate-resistant prostate cancer (128–130). MALAT1 is overexpressed and used as a prognostic biomarker in various cancer types including lung, breast, liver, pancreas, renal, colon, bladder, uterus, and cervix cancers (131, 132). On the other hand, several studies provide evidence that cancer fusion proteins can affect tumorigenicity by enhancing MALAT1 expression. For example, in multiple myeloma, an EWS-FLI1 fusion protein with an active c-MYC stimulates transcription of MALAT1 (133). Aside from fusions indirectly altering MALAT1 expression, its direct involvement in fusion events has been reported to serve as the transcriptional driver for partner protein coding gene in different cancer types:
- **MALAT1-GLI1:** MALAT1-GLI1 fusion frequently found in gastroblastoma can drive GLI1 overexpression. GLI1 encodes a transcription factor protein involved in Sonic Hedgehog signaling pathway (134). In MALAT1-GLI1, the 5′ regulatory regions of MALAT1 including its promoter, control expression of GLI1 that is lacking exons 1–4 or 5, thus leading to GLI1 overexpression which results in the production of a functional truncated protein and further upregulation of its downstream effectors (109). Further, MALAT1-GLI1 fusion is characteristic for a subtype of gastrointestinal tumor plexiform fibromyxoma (108), found in esophageal plexiform fibromyxoma (135), and was repeatedly reported in epithelioid neoplasms (110).
- **MALAT1-MVP and MALAT1-NCBP3:** In a prostate cancer mouse model, MALAT1 fusions, including MALAT1-MVP and MALAT1-NCBP3, were found to be implicated in resistance to second-generation antiandrogen cancer drugs (130). Notably, the fusion partner NCBP3 (Nuclear Cap-Binding Protein 3) is associated with nuclear–cytoplasmic transport (136), and MVP (major vault protein) is related to multidrug resistance in various malignancies (137).
- **MALAT1-TFEB:** MALAT1 is the most prevalent fusion partner in TFEB rearranged renal cells (138, 139). MALAT1-TFEB fusions occur when MALAT1 gene fuses to TFEB gene upstream of its start codon, which leads to overexpression of full-size TFEB protein (Transcription Factor EB) (140). The TFEB is a member of the micropthalmia transcription factor MiT/TFE gene family of basic helix-loop-helix leucine-zipper transcription factors. These transcription factors were shown to regulate multitude of molecular pathways well-described in carcinogenesis such as the activation of TGFβ and ETS transcription factors, mTORC1 signaling, E-cadherin expression, CD40L-dependent lymphocyte activation, insulin-dependent metabolism regulation, folliculin signaling, and retinoblastoma-dependent cell cycle arrest (138, 141). Translocations of the MiT family, particularly those involving the TFE3 and TFEB genes, have been well described in renal cell carcinoma. Since TFEB amplification is associated with a more aggressive clinical manifestation and poor outcomes for renal cell carcinoma, efforts have been made to accurately diagnose patients with TFEB translocations for improved patient management (141). Of note, the lncRNA gene NEAT1 is another frequent fusion partner in the TFEB rearranged renal cells (139). NEAT1 is normally involved in the regulation of the DNA damage repair (DDR) system (142), and it was shown to be upregulated in several cancer types (143).
- **CARMN-NOTCH2 Fusion:** In glomus tumors of the upper digestive tract, CARMN (Cardiac Mesoderm Enhancer-Associated Non-Coding RNA) ncRNA can form a fusion with NOTCH2, and the resulting chimeric transcript was linked with gain-of-function for the latter gene (144).
- **PCAT-14-ETV1 Fusion:** A prostate-specific lncRNA PCAT-14 was reported in fusion with Ets transcription factor family member ETV1. The ETS proteins regulate many target genes that modulate biological processes like cell growth, angiogenesis, migration, proliferation and differentiation (NCBI Gene ID 2115). The fusion of ETV1 and PACT-14 allowed for the androgen-regulation of ETV1 because the fusion retains the PCAT-14 promoter, which contains an androgen receptor-binding site (74, 145).
- **TTTY15 Fusions:** Among the prostate cancer patients of Asian origin, the most frequent ncRNA fusion partner was Testis-Specific Transcript, Y-Linked 15 or TTTY15 (146). TTTY15 is a lncRNA primarily expressed in the testes (NCBI Gene ID 64595). Up until now, there is no evidence about the exact function of this lncRNA. However, TTTY15 low expression was correlated with worse prognosis in non-small cell lung cancer. When investigating the mechanism, it was found that it indirectly influences the expression of TBX4, a mesenchymal transcription factor that drives the accumulation of myofibroblasts and the development of pulmonary fibrosis (147), through interacting with DNA (cyto-sine-5)-methyltransferase 3A (DNMT3A) that methylates TBX4 promoter (148). Xiao et al. explored the role of this lncRNA in prostate cancer progression and found that it acts as a competing endogenous RNA against the tumor suppressor microRNA let-7 (149). Another study by Zu et al. evaluated the clinical utility of the TTTY15-USP9Y fusion, in which TTTY15 fuses with the third exon of protein-coding USP9Y, resulting in the loss of the USP9Y protease function (150). They introduced the TTTY15-USP9Y score, which is the fold change of TTTY15-USP9Y expression normalized to that of prostate cancer–specific antigen (PSA) in urine samples of prostate cancer patients. They found that this score is independently diagnostic and can be used to indicate the necessity of biopsy as it significantly correlates with biopsy results (150).
- **BCL6-GAS5 Fusion:** In a B-cell lymphoma patient, Nakamura et al. reported a fusion of the BCL6 proto-oncogene with the non-coding GAS5 gene, leading to aberrant expression of BCL6 linked to lymphomagenesis (151).

#### 4.1.2 Oncogenic functions:

- **BCAR4-fusions:** BCAR4-fusions were frequently found in different solid tumors, and pan-cancer analysis across 32 tumor types revealed that it was the most prevalent uncharacterized downstream fusion partner in 11 cancers (123). BCAR4 is known to enhance cell migration and invasion, contributing to metastasis, chemoresistance and epithelial-to-mesenchymal transition (152–154). Importantly, BCAR4 overexpression, which has been linked to tamoxifen resistance and poor outcomes, at the same time made breast cancer cells more sensitive to lapatinib (155). The Cancer Genome Atlas (TCGA) research network reported the first fusion events in cancer involving the BCAR4 gene in cervical cancer and suggested lapatinib as a possible therapeutic option (156). The Cancer Genome Atlas (TCGA) research network reported the first fusion events in cancer involving the BCAR4 gene in cervical cancer and suggested lapatinib as a possible therapeutic option (122). Bae et al. aimed to study the mechanism of function of this fusion using *in vitro* and *in vivo* models of lung adenocarcinoma. They found that ectopic expression of CD63-BCAR4 increased the level of EGFR activated by phosphorylation, which suggests enhanced EGFR signaling. Additionally, they performed a cancer drug library screening to identify effective inhibitors of metastatic activity induced by BCAR4 fusion. Interestingly, the EGFR/HER2 inhibitors: erlotinib, canertinib, and lapatinib effectively inhibited cell migration induced by CD63-BCAR4 fusion (157). Taken together, these findings imply that EGFR/HER2 inhibitors are possible therapeutic choices for patients with BCAR4 fusion-positive lung cancer, even in the absence of EGFR mutations.

#### 4.1.3 DNA damage and cancer stemness:

- **KDM4B-G039927 and EPS15L1-lncOR7C2–1 Fusions:** In a systematic study of the lncRNA fusion landscape across cancer types using available cancer RNA databases, fusions with lncRNAs were found to be correlated with DNA damage and cancer stemness (98). The tumor-promoting properties of two of these lncRNA fusions, KDM4B-G039927 and EPS15L1-lncOR7C2–1, were validated *in vitro* on breast cancer cell lines, where their overexpression promoted proliferation (98). Although histone demethylase KDM4B is known to be linked with many tumors (158, 159) and EPS15L1 is involved in endocytosis (NCBI Gene ID: 58513), their fusion partners, G039927 and lncOR7C2–1, are less characterized. Therefore, further research is needed to understand if and how these specific fusions may drive oncogenesis.
- **PVT1 fusions:** Plasmacytoma variant translocation 1 (PVT1) is a lncRNA encoded by the human PVT1 gene located on a fragile region of chromosome 8 (8q24.21) that is prone to translocations (160). This region encodes for 52 ncRNA variants including 26 linear and 26 circular ncRNA isoforms, and six microRNAs (161). PVT1 is putatively in-volved in gene regulation through targeting of regulatory genes and modulation of miRNA expression and functioning as a competing endogenous RNA (ceRNA). Additionally, PTV1 takes part in protein interactions and somehow can activate oncogenic MYC- and STAT3- signaling (50, 162). Importantly, PVT1 is involved in different fusions found in both hematological and solid neoplasms (49). Although PVT1 itself is regarded as a potent oncogene in cancer, the function of its fusions in carcinogenesis remains unclear. One possible role of PVT1 chimeric transcripts is that they can alter transcription of target genes via enhancer-like effects (163).
- **PVT1-NSMCE2:** In leukemia cells with double minute chromosomes, which are small fragments of extrachromosomal DNA present in many human tumors, three fusion transcripts of PVT1 and the protein-coding gene NSMCE2 were identified in AML patients and cell lines of similar origin (164). NSMCE2 (Non-SMC Element 2, MMS21 Homolog) is involved in DNA double-strand break repair and the maintenance of genomic stability (165, 166). In addition, NSMCE2 has a role in maintaining telomeres, and it is involved in ensuring proper chromosome segregation during cell division (167) Thus, its overexpression may be correlated with tumor resistance to DNA damage and telomere-mediated cell cycle arrest. Notably, AML cases with 8q24 amplifications are characterized by overexpression of PVT1 and NSMCE2 (168).
- **PVT1–MYC and PVT1–NDRG1:** resulting from chromothripsis events in medulloblastoma, PVT1–MYC fusion causes MYC overexpression, leading to continuous oncogenic activity (169). **Table 1** lists MALAT1 and PTV1 fusions reported in the fusion database Chimerkb.

**Table 1:** MALAT1 and PTV1 fusions listed in Chimerkb.

Fusion pair	5’Gene Junction	3’Gene Junction	Disease	Validation method	Reference
MALAT1-GLI1	chr11:65265232 (exon:1)	chr12:57859389 (exon:6)	Wilms tumor, fibromatosis	FISH, RT-PCR	(109)
MALAT1-TFEB	Chr11:65266581 (exon:1)	chr6:41658973 (exon:3)	renal cell carcinoma	FISH, PCR	(139)
PVT1-CHD7	chr8:128902834 (exon:3)	chr8:61707544 (exon:4)	epithelioma	RT-PCR	(170)
PVT1-MYC	chr8:128902834 (exon:3)	chr8:128750493 (exon:2)	medulloblastoma	RT-PCR	(169)
PVT1-NBEA	chr8:128806778 (exon:1)	chr13:35615069 (exon:2)	multiple myeloma	FISH, RT-PCR	(171)
PVT1-NDRG1	chr8:128806778 (exon:1)	chr8:134262786 (exon:10)	medulloblastoma	Sanger sequencing	(169)
PVT1-NSMCE2	chr8:128806778 (exon:1)	chr8:126114558 (exon:3)	leukemia, acute myeloid leukemia (AML)	FISH, RT-PCR	(164)
PVT1-WWOX	chr8:128806778 (exon:1)	chr16:79245504 (exon:9)	multiple myeloma	FISH, RT-PCR	(171)

### 4.2 Fusion circRNAs

Fusion circRNAs (f-circRNAs) can result through back-splicing when chromosomal translocation events bring complementary intronic sequences up- and down-stream of the breakpoint (172, 173). Alternatively, circRNAs can be generated from transcription read-through of adjacent genes, usually referred to as read-through circRNAs (174). f-circRNAs resulting from chromosomal translocations can be functionally relevant oncogenes and may promote cancer development. In a mouse model of acute myeloid leukemia (AML) expressing the MLL–AF9 fusion protein, f-circRNA produced from the same fusion gene was shown to promote leukemia progression (102). Guarnerio et al. investigated the oncogenic role of f-circRNAs generated by gene fusions in leukemia, among them the f-circRNA named f-circM9, generated from the MLL-AF9 gene fusion in AML. This gene fusion is one of the mixed lineage leukemia 1 (MLL1) gene chromosomal translocations that gave rise to the MLL-rearranged acute myeloid and lymphoblastic leukemia subset (175). The MLL-AF9 fusion joins the AF9 protein, found in the super elongation complex (SEC) that facilitates transcriptional elongation, with MLL. This fusion functions by abnormally attaching the DNA-binding protein SEC to MLL, thus causing the incorrect expression of MLL target genes. Be-sides, AF9 is present in the DOT1L complex (DOTCOM), causing aberrant modifications (H3K79me2) on actively expressed genes. Therefore, blocking DOT1L could be a promising treatment strategy for MLL-rearranged leukemia (176).

Furthermore, f-circM9 has been demonstrated to have pro-proliferative and pro-oncogenic properties, as well as to enhance cell viability and resistance to chemotherapy (102). Further, authors identified f-circPR originated from PML-RARα fusion gene in promyelocytic leukemia. Both f-circM9 and f-circPR were found to stimulate the activation of the PI3K and MAPK signaling pathways, thus exerting pro-proliferative and proto-oncogenic activities. However, while testing the leukemia progression effects of f-circM9 *in vivo*, this f-circRNA alone appeared to be not sufficient to trigger tumorigenesis and probably needs the presence of additional oncogenic stimuli (102).

Apart from leukemia, Guarnerio et al. revealed the expression of f-circRNAs originating from EWSR1-FLI1 and Echinoderm Microtubule-associated protein-Like 4-Anaplastic Lymphoma Kinase (EML4-ALK1) linked to Ewing sarcoma and lung cancer respectively, indicating that f-circRNA expression is not exclusive to leukemia cases (102).

In non-small cell lung cancer (NSCLC), Tan et al. identified two f-circRNAs, F-circEA-4a and F-circEA-2a, generated from the EML4-ALK fusion gene variant 3b (112, 177). F-ciricEA-4a, harboring “AAAA” motif at junction site, was investigated for its role in cancer, where it showed oncogenic activity independently from the onco-genic fusion transcript and protein EML4-ALK, hence contributing to tumor development. F-circEA-4a stimulates cell migration and invasion in H1299 and A549 cell lines not harboring the EML4-ALK translocation and hence do not express the endogenous fusion protein. F-circEA-4a has been proposed as a potential liquid biopsy biomarker since it is detectable in the plasma of EML4-ALK-positive NSCLC patients, thus aiding in the diagnosis of EML4-ALK-positive NSCLC patients to guide targeted therapy with approved ALK inhibitors (177). Furthermore, F-circEA-2a was expressed in the cyto-plasm of lung cancer cells promoting migration and invasion (112).

Additionally, in NSCLC Wu et al. reported two f-circRNAs: F-circSR1 and F-circSR2, generated from SLC34A2-ROS1 fusion gene (178) These f-circRNAs were shown to promote lung cancer cell migration with small effect on proliferation independently form SLC34A2-ROS1 fusion protein. Moreover, Huo et al. have recently reported a F-circRNA, F-circEA1, generated from the EML4-ALK fusion gene variant 1. Their work demonstrated that F-circEA1 promotes cell proliferation, metastasis and invasion and also regulates cell proliferation and apoptosis via the ALK downstream signaling pathway (179). These roles of F-circEA1 were confirmed through CCK8 and transwell assays. Similar effects were observed in A549 and SPCA1 cells, which are non-small cell lung cancer cell lines that do not carry the EML4-ALK1 fusion gene. Further, interfering with F-circEA1 led to cell cycle arrest, increased cell death, and in-creased sensitivity to the drug crizotinib. Moreover, in live models, F-circEA1 interference slowed down tumor growth and notably reduced EML4-ALK1 protein expression in the implanted tumors. Therefore, F-circEA1 could be classified as a proto-oncogene that regulates cell growth and death by impacting the expression of its parental gene, EML4-ALK1, and its downstream pathway in NSCLC. We highlighted selected cases of f-circRNAs and their putative functions in cancer on **Table 2**.

**Table 2:** Examples of f-circRNAs and their parental fusion genes in human cancers.

Cancer type	Fusion gene	f-circRNA	Function	Therapeutic potential	References
NSCLC	EML4-ALK1	F-circEA1	Cell proliferation and apoptosis regulation	ND	(179)
	EML4-ALK3b	F-circEA-4a	Promotion of cell migration and invasion	plasma diagnostic biomarker	(177)
	EML4-ALK3b	F-circEA-2a	Promotion of cell migration and invasion	ND	(112).
	SLC34A2-ROS1	F-circSR1 and and F-circSR2	Promotion of cell migration	ND	(178)
Ewing Sarcoma	EWSR1-FLI1	f-circEF1	ND	ND	(102).
Acute lymphoblastic (ALL) and acute myeloid leukemia (AML)	MLL-AF9	f-circM9	Proliferative and oncogenic activity in immortalized mouse cell line	ND	(102, 180).
Acute Promyelocytic leukemia	PML-RARα	f-circPR	proliferative and oncogenic activity in immortalized mouse cell line	ND	(102).

CircRNAs were shown to be relatively stable and having a half-life in general longer than that of their linear counterparts by at least 2.5 times (181), Furthermore, circRNAs were abundant in serum exosomes in colorectal cancer (174, 182), thus they potentially can be detected in minimally invasive liquid biopsy settings (102, 183). Guarnerio et al. analyzed a few chromosomal translocations in a variety of cancer types and found that about 50% of these chromosomal translocations gave rise to specific f-circRNAs, which again highlights their possible oncogenic and/or diagnostic potential (55). In contrast, a more recent analysis of MiOncoCirc data, encompassing 17 cancer sequencing datasets, did not identify any fusion circular RNAs (f-circRNAs) resulting from chromosomal rearrangements. Instead, they unveiled a new class of circular transcripts termed read-through circular RNAs, which include exons from two neighboring genes on the same strand. These read-through circRNAs were frequently found across different cancer types and were also abundant in the normal tis-sues (184).

## 5 Discussion

Approximately 76% of the human genome is being actively transcribed according to the ENCODE project findings, with only ~1% fraction of the genome that encodes proteins (185, 186) The significance of ncRNAs is increasingly acknowledged particularly in the last decade, after being previously considered mostly as “transcriptional noise”. The majority of ncRNAs were discovered only recently and remain largely underexplored (50). Numerous studies aimed to investigate the role that ncRNAs may play in association with the chromosomal rearrangements in cancer. They are not only affect-ed by the products of chromosomal translocations and associated fusion proteins or their downstream effectors (47), but chromosomal translocations can also directly disrupt ncRNA host genes, thus causing their dysregulated expression (187, 188).

Until now there are rather sporadic reports of ncRNA-involved fusions, making them an underestimated class of gene fusions despite its potential biological and clinical implications across solid tumors. This highlights the growing need for their standardized and comprehensive documentation. When analyzing fusion transcripts, applying an ncRNA-centered approach can significantly enhance our understanding of the relevance and impact of ncRNA genes in fusion events (100). This method helps with the identification and characterization of ncRNA fusions, which often go unnoticed when using traditional protein-coding centric analysis. By focusing on ncRNAs, researchers can uncover the roles these molecules play in regulating gene expression, contributing to tumorigenesis, and interacting with their host genes. With more ncRNA fusions being detected (48), a functional characterization of ncRNA-involving fusions across cancer types should be performed, as well as the systematic analysis of their mechanisms implicated in carcinogenesis.

In the era of personalized medicine and precision oncology, ncRNAs and fusion genes emerge as important diagnostic factors and/or possible therapeutic targets. The genomic events that led up to the progression of tumor are highly diverse among individuals even for the same cancer type. With the continual expansion of clinical sequencing data and the pharmaceutical arsenal, each patient’s therapy is anticipated to become increasingly customized based on decoding its individual tumor biology. In this context, targeting ncRNAs and their fusions was predicted to become effective in many clinical scenarios. For example, several clinical trials with therapies affecting expression of target miRNAs were recently initiated in cancer settings (50). Given that most ncRNAs act in a cell-specific environment, identifying the main misregulated ncRNAs for a certain disease can lead to the identification of ncRNA candidates for therapeutic targeting (189). Thus, a better knowledge of the function and diversity of chimeric RNAs may raise the chance of successful RNA-based therapy application in many cancers.

With the introduction of low-cost high-throughput sequencing methods including whole-genome sequencing, long-read sequencing, and single-cell RNA sequencing, we can now uncover an unprecedentedly high number of fusion genes with ncRNAs. We believe that the ongoing research in this field has the potential not only to unravel new mechanisms of cancer development and progression but can also lead to significant advancement in cancer diagnostic and therapeutic options.

## Author contributions

TM: Visualization, Writing – original draft, Writing – review & editing. MZ: Writing – review & editing. MS: Writing – review & editing. AB: Conceptualization, Writing – original draft, Writing – review & editing.
